# Effects of exercise on symptoms of anxiety, cognitive ability and sick leave in patients with anxiety disorders in primary care: study protocol for PHYSBI, a randomized controlled trial

**DOI:** 10.1186/s12888-019-2169-5

**Published:** 2019-06-10

**Authors:** Jenny Nyberg, Malin Henriksson, N. David Åberg, Alexander Wall, Robert Eggertsen, Maria Westerlund, Louise Danielsson, H. Georg Kuhn, Margda Waern, Maria Åberg

**Affiliations:** 1 Center for Brain Repair and Rehabilitation, Institute of Neuroscience and Physiology, Sahlgrenska Academy, University of Gothenburg, Gothenburg, Sweden; 2 Neurology Clinic, Sahlgrenska University Hospital, Region Västra Götaland, Gothenburg, Sweden; 3 Department of Public Health and Community Medicine/Primary Health Care, Institute of Medicine, Sahlgrenska Academy, University of Gothenburg, Box 454, SE-405 30 Gothenburg, Sweden; 4 Department of Internal Medicine, Institute of Medicine, Sahlgrenska Academy, University of Gothenburg, Gothenburg, Sweden; 5 Department of Internal Medicine, Sahlgrenska University Hospital, Region Västra Götaland, Gothenburg, Sweden; 6 Region Västra Götaland, Närhälsan, Gothenburg, Sweden; 7 Department of Health and Rehabilitation, Institute of Neuroscience and Physiology, Sahlgrenska Academy, University of Gothenburg, Gothenburg, Sweden; 8 Angered Hospital, Region Västra Götaland, Gothenburg, Sweden; 9 Center for Stroke Research and Neurocure Cluster of Excellence, Charité – Universitätsmedizin Berlin, Berlin, Germany; 10 Department of Psychiatry and Neurochemistry, Institute of Neuroscience and Physiology, Sahlgrenska Academy, University of Gothenburg, Gothenburg, Sweden; 11 Psychosis Clinic, Sahlgrenska University Hospital, Region Västra Götaland, Gothenburg, Sweden

**Keywords:** (n=3-10), Anxiety, Exercise, Cognitive function, Dose-response, Intervention

## Abstract

**Background:**

Anxiety disorders are common and associated with reduced quality of life, impaired physical and mental health and an increased economic burden for society. While evidence exists for the effectiveness of exercise treatment for depression, there is a need for high-quality randomized clinical trials (RCT) with a focus on anxiety disorders. Further research is also warranted regarding outcomes of cognitive function, other health-related variables, dose-response effects, work ability and potential mechanisms.

**Method/design:**

Using a parallel, RCT design with three assessment points (baseline, post-intervention and one-year follow-up), we aim to assess the effect of a 12-week exercise intervention in primary care patients with anxiety disorders (*n* = 180), diagnosed using the Mini International Neuropsychiatric Interview (M.I.N.I; Swedish version 6.0.0d DSM-IV). Participants are randomly assigned to three physical exercise groups: one low-intensity training group, one moderate- to high intensity training group and one control non-exercise group. Assessments include measures of anxiety symptoms, cognitive function, physical health variables such as cardiovascular fitness, sick-leave and levels of hormones/cytokines in blood samples.

**Discussion:**

Findings from this study will provide novel insights regarding the effects of exercise treatment on not only anxiety symptoms but also other outcomes including mental and physical health, cognitive function, dose-response effects, work ability/sick leave and on biomarkers that may help explain underlying mechanisms.

**Trial registration:**

The trial was registered at ClinicalTrial.gov NCT03247270 August 8, 2017.

**Electronic supplementary material:**

The online version of this article (10.1186/s12888-019-2169-5) contains supplementary material, which is available to authorized users.

## Background

Anxiety disorders are the most common mental disorders [1], with global prevalence figures ranging from 4 to 25% [2]. Data from the US indicate that one out of three will be affected by an anxiety disorder at some point during lifetime [1]. In a national survey of the Swedish adult population, 36% reported having anxiety issues [3] and lifetime prevalence for anxiety disorders in Sweden is estimated to be 25% [4]. In Sweden, it has been estimated that 70% of individuals who seek help for symptoms of anxiety and depression initially present in primary care [5] and costs for mental illness within primary care are on the rise [6].

Anxiety disorders substantially reduce quality of life and daily functioning [7] and are, among mental disorders, the second leading cause of years lived with disability [8]. They are also associated with elevated risks of cardiovascular disease [9] and premature mortality [10]. We have previously shown that anxiety disorders are associated with increased risk of future marginalization [11] as well as elevated mortality [12], emphasizing the importance of finding effective treatment strategies. Individuals with anxiety disorders frequently suffer from attentional problems, such as being easily distracted and unable to focus on ongoing tasks [13]. Moreover, impairments in cognitive function, including executive function and working memory, are often observed, which can be both a consequence and a symptom of anxiety [14–16]. Taken together, anxiety disorders place a considerable emotional, social, health-related and financial burden on both the individual and society.

The standard treatments for anxiety disorders include cognitive-behavioural therapy and pharmacological treatment [17]. Although these approaches often are effective in reducing anxiety symptoms, they may also be associated with treatment barriers and drawbacks. For example, nearly one third of patients do not respond to pharmacological treatment which may be associated with adverse side effects and noncompliance [17]. Patients may not wish to follow cognitive-behavioural therapy due to a reduced willingness to commit to treatment and stigmatization issues [18]. Collectively, these findings call for alternate complementary or stand-alone approaches to the treatment of anxiety disorders. Moreover, given the known association between anxiety disorders and cardiovascular disease, it is also essential to develop interventions that may target not only mental but also physical health problems in people with anxiety disorders. Exercise may represent an alternative, affordable and accessible treatment option for individuals with anxiety, both by itself and together with standard treatments. For clarification, *exercise* is a subset of physical activity that is planned, structured, repetitive and has a purpose of improving or maintaining physical fitness and *physical activity* is defined as any bodily movement produced by skeletal muscles that result in energy expenditure [19]. *Physical fitness* on the other hand is a set of attributes that are either health- or skill-related, including cardiovascular fitness and muscle strength.

Observational studies report an inverse association between physical activity and anxiety symptoms [20] and there is strong evidence for exercise in the treatment of depression [21]. Despite these findings, relatively little research has been focused on exercise in the treatment of anxiety. Nonetheless, meta-analyses of randomized controlled trials (RCTs) studying anxiolytic effects of exercise interventions in individuals with and without an anxiety diagnosis suggest that exercise is an effective treatment on its own, as well as combined with other therapies [22–26]. However, most of the interventions studies included in these meta-analyses are at high risk of bias. High-quality RCTs are needed to gain further understanding of the effects of exercise in treatment of anxiety [24] and to develop optimal exercise protocols.

Key gaps in the literature remain regarding the effects of exercise on other mental and physical health variables in patients with anxiety disorders including cognitive function, cardiovascular fitness, optimal exercise protocols, work ability/sick leave as well as the underlying mechanisms of the effect of exercise on anxiety symptoms. These are all issues that the current study aims to address. Given that cognitive function can be improved by exercise [27], the current study includes tests of executive function, creativity, response inhibition and cognitive flexibility. We will also evaluate the intervention effects on cardiovascular fitness, muscle strength, BMI and blood pressure. Further understanding of dose-response relationships is important for establishing optimal protocols for exercise treatments in patients with anxiety, instead of only having to rely on general public health guidelines. Since dose-response effects of exercise have been minimally researched and studies of dose-response are often inconclusive [22, 23], we include two intervention groups with separate intensity levels of exercise training. It is also highly relevant to evaluate the effects of exercise on work ability in patients with anxiety disorders, since reducing medical and sick-leave costs for anxiety disorders would liberate health care resources to be used elsewhere in an economically strained health care system. In order to gain further understanding regarding mechanistic aspects, treatment effect and prognosis, we will also investigate protein levels of trophic factors in blood samples, through which exercise may reduce anxiety. A broad spectrum of possible mechanisms exists, explaining the effect of exercise on anxiety including neurotransmitter, neuromodulator and psychological mechanisms [28], but the precise mechanism remains unclear.

We here propose an RCT study with varying intensity of exercise training in patients with anxiety disorders. To the best of our knowledge, this multifaceted study is the first study of its kind and it has the potential to provide novel and important contributions to existing scientific knowledge regarding how exercise may influence symptoms of anxiety, cognitive function, general health and work ability in patients with anxiety disorders.

### Research aims and hypothesis

The primary aim is to investigate the effects of an exercise intervention on anxiety symptoms, cognitive function, physical health and quality of life and work ability in primary care patients who fulfil criteria for anxiety disorders. A secondary aim is to analyse any potential correlation of factors in blood samples (hormones/cytokines) with anxiety symptoms and function data and to study possible dose-response effects in patients assigned to exercise of different intensities. Another secondary aim is to study mediating or moderating effects of cardiovascular fitness or other comorbidities and to analyse possible differential effects in sub-groups of participants (different initial cardiovascular fitness levels, ages and gender). In addition, health-economic analyses will be performed concerning health-related quality of life effects associated with the intervention.

Our main hypothesis is that an exercise intervention will reduce symptoms of anxiety and the number of days on sick leave, improve cognitive function and physical health variables. An additional hypothesis is that the effect will be larger for high-intensity exercise than for low-intensity.

## Methods/design

### Study design

**Fig. 1:**
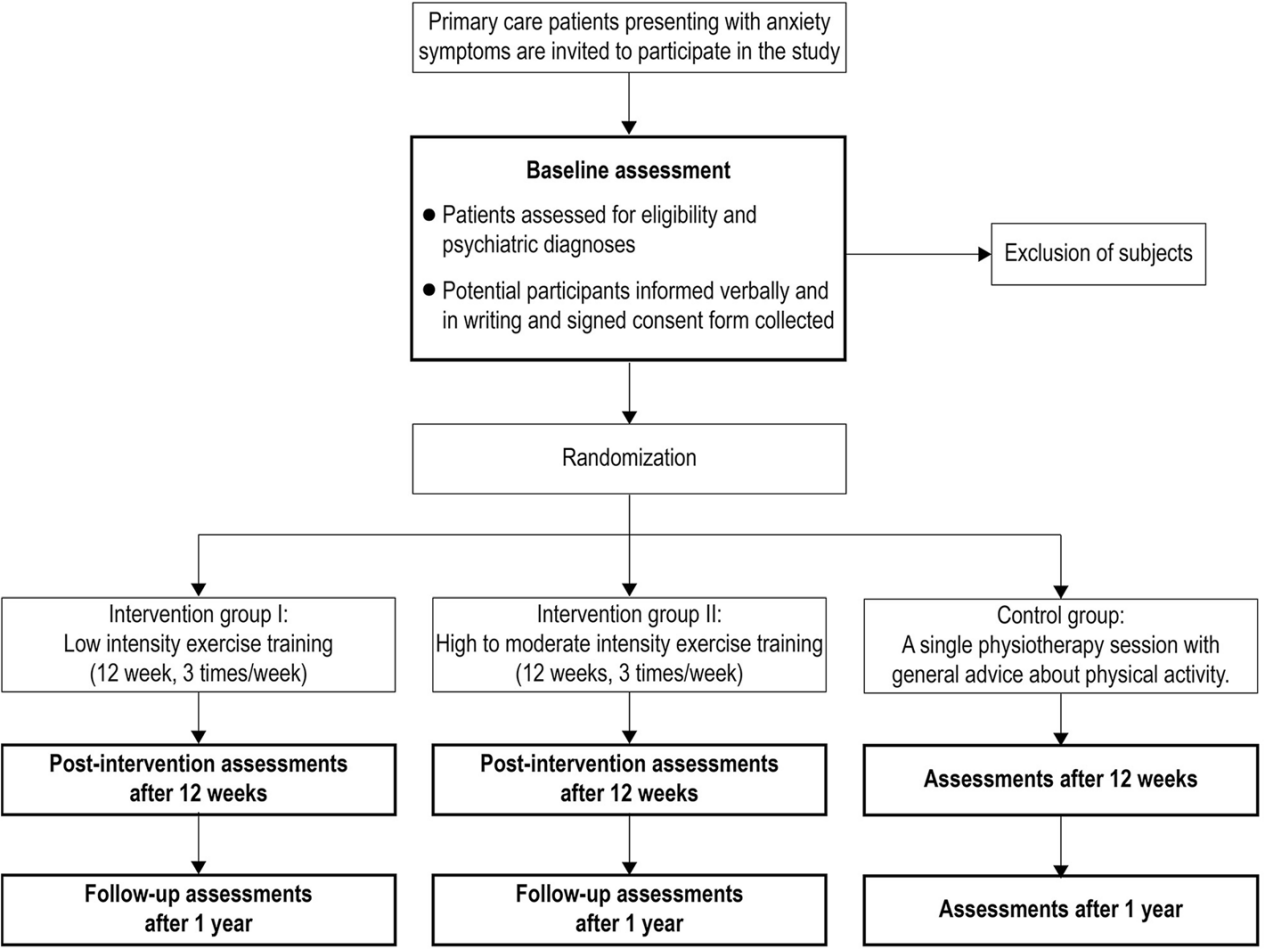
Flow chart of study design

This is a randomized, parallel, controlled superiority clinical intervention study with three assessment points (baseline, post-intervention and one-year follow-up). The study is ongoing and was registered under ClinicalTrial.gov NCT03247270 on August 8, 2017 (https://clinicaltrials.gov/ct2/show/NCT03247270). The protocol was prepared in accordance with the SPIRIT 2013 statement. [29] Participants are randomly assigned to an exercise program of one low-intensity training group, one moderate- to high intensity training group and one control non-exercise group, with 1:1:1 allocation. Assessments and interventions are conducted at Primary Care Rehab (Närhälsan Primary Care) in Gothenburg, Sweden. Figure 1 shows the study design of the trial.

### Participants

Individuals who seek help for anxiety issues at six primary care centres in southwest Sweden (Region Västra Götaland and Region Halland) are recruited. The patients are informed by their general practitioner (GP) or psychologist about the possibility of study participation and those expressing interest are provided with the study physician’s phone number. Recruitment started in May 2017 and will continue until March 2020. Potential participants are then contacted by the study psychiatrist or nurse, who have no healthcare provider relationship to the patient.

Diagnoses and comorbidities are then determined by the study psychiatrist using the Mini International Neuropsychiatric Interview (M.I.N.I; Swedish version 6.0.0d DSM-IV), a short structured diagnostic interview with high reliability and validity [30]. Patients aged 18–65 diagnosed with anxiety disorders, including panic syndrome (F41.0), generalized anxiety (F41.1), mixed anxiety- and depression (F41.2 and F41.3), as well as anxiety NOS (F41.9) are included in the study.

Exclusion criteria include physical difficulties that preclude participation in the present exercise program, pathological electrocardiogram (ECG) including significant Q waves, ST-T changes, atrial fibrillation and left bundle-branch block, use of beta blockers, pregnancy, as well as previous psychiatric illness including bipolar disorder and psychotic disorders, ongoing substance abuse, ongoing burnout syndrome, and increased risk of suicide as assessed by the GP. An additional exclusion criterion is baseline physical activity level exceeding one exercise occasion per week. Individuals with and without ongoing treatment with psychoactive medication are included. Supportive contact, for example with a health center nurse, is not an exclusion criterion but individuals with ongoing psychotherapy are not included unless, after consultation with their GP, they can take a break in therapy for the duration of the 12-week intervention. Participant retention and adherence is promoted by exercise encouragement from the study physiotherapists, the possibility to receive personal results after study completion and reminders for follow-up assessment via letter, telephone and text message. The reasons for all eventual non-adherence and non-retentions will be documented.

### Intervention

Using computer generated randomisation (http://www.randomizer.org), patients are randomized into three groups: 1) Intervention I: 12-week exercise program with low-intensity training 3 times per week for 45 min; 2) Intervention II: 12-week exercise program with moderate to high-intensity training 3 times per week for 45 min; 3) Control group: a single physiotherapy session with general advice about physical activity. Participants are then informed by phone if they belong to an intervention group or the control group. Both intervention groups include cardiorespiratory and resistance training, and participants in group II are also encouraged to perform an additional running session per week. Participants in the control group are encouraged to start exercising after the study is completed and also receive a 3-months membership at a training facility, but are discouraged of actuating other exercise programs during the trial. All three groups self-report physical activity during the study. For participants in the intervention groups, the study physiotherapists designed individualized exercise programs during a one-to-one session with the patients on one occasion. The exercise program is designed as a circuit training with 12 stations, repeated twice (45 s workout, 15 s for transportation between stations and 1 min rest between the two laps). It also includes 10 min of warm-up exercises and 5–10 min of cool down and stretching exercises. Cardiorespiratory exercises include step-ups, lunges, jump rope, burpees, step touches side-to-side and step-touches on step board. Resistance training exercises include squats, abdominal plank position, hip lifts, crunches, row exercises and push-ups. Participants in intervention group I and II exercise separately in group sessions in the gym with the physiotherapist according to a predetermined schedule. A pulse-watch and the Borg RPE scale are used to monitor level of exertion to ensure appropriate exercise intensity. For intervention group I, the intensity level corresponds to 1.5–2.9 metabolic equivalents (METs), a Borg rated perceived exertion (RPE) of 10–14 and a maximal heart rate of 40–59%. The corresponding figures for intervention group II are 3.0–8.9 METs, Borg RPE 12–17 and 60–94% of maximal heart rate. Patients who are unable to participate in a session perform replacement exercises on their own.

### Blinding

Researchers involved in data assessments (outcome assessors, study physicians, study psychologists, study psychiatrists, statistician and data collector) are blinded to the patients’ intervention group. The only personnel aware of the treatment groups are the study physiotherapists who randomize the participants to the groups, code them, and also deliver the exercise interventions, but they are not involved in any data analyses. For half of the included patients, participation in low or high intensity training group was double-blind to both study researchers and patients: for the other half of the patients, training intensity group was only blind to the study researchers (the patients were aware of what treatment group they belonged to). Comparison of these two study halves will enable us to determine if blinding procedures reduce result bias. Final unblinding will occur after all assessments are performed and data collected at the 1-year follow-up (June 2021).

### Assessments

Assessments occur at baseline, post-intervention (after 12 weeks) and at one-year follow-up (Table 1). Participants will be assessed by the study physician, psychologist, psychiatrist and physiotherapist.

**Table 1:** Measures, methods and assessment points used in the study

Variable	Measure	Method	T0 Baseline	T1 12 weeks	T2 1 year
Psychiatric diagnosis	M.I.N.I.	Interview	X	X	X
Anxiety symptoms	BAI	Self-report	X	X	X
Depression symptoms	MADRS	Self-report	X	X	X
Psychoactive drugs	Questionnaire	Self-report	X	X	X
Blood pressure	MmHg	Objective assessment	X	X	X
BMI	Kg/m^2^	Objective assessment	X	X	X
Alcohol use	AUDIT	Self-report	X	X	X
Quality of life	EQ-5D	Self-report	X	X	X
Cognitive function	WAIS-IV (d.s., b.d., m.r.)	Objective assessment	X	X	X
Cognitive function	D-KEFS (d.f.)	Objective assessment	X	X	X
Cardiovascular fitness	Åstrand’s ergometer test	Objective assessment	X	X	X
Muscle strength	One-leg-rising-test	Objective assessment	X	X	X
Sick leave	Questionnaire	Self-report	X	X	X
Hormones/cytokines	S-IGF-1, S-BDNF, S-VEGF, CRP	Blood samples	X	X	X

*M.I.N.I.* Mini International Neuropsychiatric Interview, *BAI* Beck Anxiety Inventory, *MADRS* Montgomery Åsberg Depression Rating Scale, *BMI* Body mass index, *AUDIT* Alcohol Use Disorders Identification Test, *EQ-5D* EuroQoL-5 Dimension Questionnaire, *WAIS-IV* Wechsler Adult Intelligence Scale, 4th edition, *d.s*. digit span, *b.d*. block design, *m.r.* matrix reasoning, *D-KEFS* Delis-Kaplan Executive Function System, *d.f*. design fluency, *S-IGF-1* serum insulin-like growth factor 1, *S-BDNF* serum brain-derived neurotrophic factor, *S-VEGF* serum vascular endothelial growth factor, *CRP* high sensitivity C-reactive protein

#### Mental health variables

Psychiatric diagnoses, determined by the study psychiatrist using M.I.N.I., are applied at initiation, at 12-weeks post-intervention and 1-year follow-up. Severity of perceived symptoms of anxiety and depression are assessed using the established psychiatric self-assessment scales Beck Anxiety Inventory (BAI) [31] and Montgomery Åsberg Depression Rating Scale (MADRS) [32]. Patients report use of psychoactive prescription drugs, including dosage and duration at each assessment.

#### Cognitive function

The Wechsler Adult Intelligence Scale, 4th edition (WAIS-IV) is used to measure executive functions and perceptual reasoning [33]. The *digit span forward*, *backward* and *sequencing* subtests were chosen to measure executive function, working memory, attention and mental manipulation. The *block design* and *matrix reasoning* subtests were chosen to measure visual-spatial processing and nonverbal problem-solving skills [33].

The Delis-Kaplan Executive Function System (D-KEFS) is a standardized, non-verbal psychomotor test battery assessing executive functions in individuals between 8 and 89 years old [34]. The subtest *Design fluency* resembles the cognitive chain required in daily life to generate novel responses, while maintaining focus on a desired goal, and measures processes such as creativity, response inhibition, and cognitive flexibility [34, 35]. The *Design fluency* subtest consists of three conditions, all performed using a pen and paper where the participant has to bind together dots and make as many novel shapes possible during 60 s. The participant needs to use working memory, inhibition and scanning skills to remember previously drawn shapes, inhibit the response to repeat shapes and scanning skills and creativity to find novel solutions to complete the assignment. The tasks appear in order of increasing difficulty, and the final trial includes a cognitive set-shifting component. D-KEFS has been used in both clinical and research settings showing good reliability and validity for measuring executive functions [35, 36].

#### Physical health variables

Blood pressure, weight and height are measured and body mass index (BMI) is calculated for each patient. Alcohol use is assessed using the Alcohol Use Disorders Identification Test (AUDIT) [37]. Patients self-report smoking, diet habits, concurrent illnesses, usage of prescribed drugs and physical activity in a questionnaire designed by the research team (Additional file 1). General health and perceived quality of life are measured using the EuroQoL-5 Dimension Questionnaire (EQ-5D), a standardized instrument measuring health related to five dimensions: mobility, self-care, usual activities, pain/discomfort and anxiety/depression [38].

#### Cardiovascular fitness

Maximal oxygen uptake capacity (VO2max) is estimated using Åstrand’s submaximal ergometer test and serves as a measure of cardiovascular fitness [39]. The test is performed by the physiotherapists at Primary Care Rehab. Participants are told to refrain from vigorous activity the day before the test, to avoid a heavy meal within three hours and smoking/snuff use one hour prior to the test and also to avoid stress during the test day. The ergometer is calibrated for each participant and heart rate is corrected for sex and age. Predicted VO2max is calculated in accordance with the nomogram described by Åstrand and Ryhming [39].

#### Muscle strength

Muscle strength is measured using a standardized test of maximum number of one-leg rises from sitting on a chair (45 cm high) during 1 min [40]. The patient is told to keep the arms crossed and to hold the other leg extended without touching the floor. The test is performed with full muscle control and at constant speed without adding arm or trunk movements.

#### Work ability

Patients self-report work ability, weekly number of work hours and if they are on sick-leave in a questionnaire (Additional file 2).

#### Blood samples

Blood samples are taken by a biomedical analyst to investigate any potential correlation of the following hormones/cytokines with anxiety symptoms and function data: S-insulin-like growth factor 1 (IGF-1), S-brain-derived neurotrophic factor (BDNF), S-vascular endothelial growth factor (VEGF) and high sensitivity C-reactive protein (CRP). Total accumulated blood volume is 15 ml per individual. The blood samples are coded and stored in a separate, locked freezer (− 70 degrees) at Sahlgrenska University Hospital, Gothenburg. All analyses will be performed in collaboration with the central laboratory at Sahlgrenska University Hospital, Gothenburg. After study completion, the samples will be destroyed. The Swedish Health and Social Care Inspectorate (IVO), a government agency under the Ministry of Health and Social Affairs, has been notified of the establishment of a biobank (No 946).

### Sample size calculation/power calculation

A power analysis was performed by a statistician prior to study initiation. We have calculated, based on other studies [41, 42], that an effect size of 0.5 difference between the groups is clinically relevant, which equates to a 5-point difference between the groups on the BAI scale. Standard deviations from a previous study using BAI as the outcome measurement were in the interval of 5–10 [43]. We use SD 9 for our calculations. Based on these assumptions, we should include 60 patients in each of the treatment arms to reach 80% power at the 5% significance level. In order to achieve an adequate number of study participants, we will initially recruit 25% more subjects than required.

### Statistical analysis

Data will be presented using descriptive statistics, including the number of observations, means and standard deviations for continuous variables. For categorical variables, frequencies and percentages will be presented and data analysed using Chi-squared tests. Based on plotted histograms (confirming that the data may be considered as approximately normally distributed), continuous data will be analysed using a univariate general linear model (ANCOVA). In this model, treatment will be used as a categorical independent variable and baseline levels as covariates. Data will be presented as changes of means with corresponding 95% confidence intervals, standard deviations and *p*-values from group comparisons. The categorical outcome “treatment responsive” will be analysed using Chi-squared tests. A p-value below 0.05 will be considered statistically significant for all analyses. All statistical analyses will be performed by a statistician using SAS version 9.4 (SAS Institute, Cary, NC).

#### Drop-out analyses

Drop-out analysis is a type of sensitivity analysis where we aim to examine differences between drop-outs and participants of all study groups concerning several descriptive variables (age, gender, BMI, cardiovascular fitness, perceived anxiety level, cognitive function, comorbidity) in order to investigate if the incidence of drop-outs is selective or random.

Outcome data collected prior to an eventual later study discontinuation will be included in the analyses i.e. a participant lacking data from the 1-year follow-up will still have data from the 12-week post-intervention.

#### Health-economic analyses

Health-economic analyses will be performed using primary data generated within the project concerning health-related quality of life effects associated with the intervention and information about healthcare resources utilized. A health-economic model that depicts the clinical trial will be constructed and used for computing cost-utility measures. Moreover, the model will be constructed for performing extensive probabilistic sensitivity analyses [44, 45].

### Project timeline

**Fig. 2:**
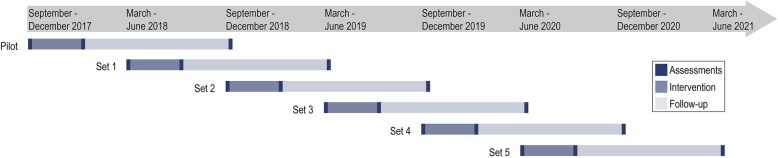
Study timeline

Recruitment started in May 2017 and will continue until March 2020. The trial is divided into one pilot set and five trial sets and the estimated primary completion date is June, 2021 (Fig. 2).

### Ethical considerations

The study is approved by the regional Ethics Committee in the Gothenburg, Sweden, National Board of Health prior to start (300–16) prior to start.

Interested patients are informed verbally and in writing about the aims and the study design including randomization, the importance of active participation for reliable data but also about the possibility to withdraw from the study at any time without disadvantages. Each participant has to sign a statement of informed consent, delivered by the study physician, before study inclusion.

All data collected will be coded and anonymized. A key connecting serial numbers with personal identification numbers will be stored and locked away. All analyses will be performed on coded data presented on group levels. The database will be password-protected, encrypted and stored for 10 years in a locked, fireproof archive at Gothenburg University, designed for such purposes. All participating researchers will have access to the database. Collected blood samples will be destroyed after the completion of the study. A Data Protection Officer (DPO) has been assigned and reported to the Swedish Data Protection Authority.

The current research team has previously performed an intervention study on the effects of exercise on depressed patients [46] and hence anticipates low risk of complications. A small risk for injury while exercising exists, such as a sprained ligament or a strained muscle. In order to minimize the risk for injuries, exercise programs are developed and modified according to the ability of each patient by experienced primary care physiotherapists. Moreover, ECG is monitored before the intervention and individuals with a pathological ECG will be excluded, which will minimize the risk for cardiac side-effects during exercise.

In order to reduce the risk for worsening mental health or symptoms during the study we have a) assessed eligibility for study participation by a physician; b) excluded patients with heightened suicide risk; c) requested participants to contact research personnel if they feel worse; d) as intervention personnel, only included persons with knowledge regarding symptoms of worsening of anxiety and/or depression. If a patient experience worsening of symptoms, the subject will be excluded from the study and put in contact with the treating physician or psychologist. The study physician and physiotherapists will continuously inquire about and record any signs of worsening of mental health symptoms or side-effects.

Another experience from our previous intervention study on depression was the disappointment of being randomized to the control group. Therefore, the patients in the control group will have a single session with a physiotherapist and general advice about physical activity according to public health recommendations as well as a 3-month membership at a training facility for after study completion. Such a control condition, was chosen to potentially feel meaningful and inspiring to the participants. [47] A passive control group such as a wait-list design or treatment as usual are associated with negative influences on study results for nonpharmacological treatments, including a possible overestimation of the intervention effects and limited blinding possibilities. [48] Instead of an active control with an alternative behaviour, the current study has two separate interventions groups with different exercise intensities, a design that allows for comparison effectiveness depending on exercise dose as well as permits blinding for participants. All study participants will be informed about the results from the study, as will all participating professionals and the general medical community. Results will be published in peer-reviewed medical journals and results will be disseminated regardless of the magnitude or direction of effect. When writing manuscripts, ICMJE (International Committee of Medical Journal Editors) guidelines regarding authorship will be followed.

## Discussion

This is an extensive intervention study (RCT) exploring the effects of exercise training in primary care patients with anxiety. The unique study design with several strengths will provide novel insights and include outcome measures not only for anxiety symptoms, but also for a broad range of areas including mental and physical health, cognitive function, dose-response effects, work ability/sick leave and underlying mechanisms. If this exercise trial proves beneficial, this study will provide new directions for primary care service regarding prevention and treatment for persons with anxiety disorders.

## Additional files

Additional file 1: Questionnaire PHYSBI. (PDF 111 kb)

Additional file 2: Work ability PHYSBI. (PDF 81 kb)

## Data Availability

The database generated for the current study is not publicly available for ethical reasons but data on group level are available from the corresponding author on reasonable request.

## Abbreviations

AUDIT: Alcohol Use Disorders Identification Test
BAI: Beck Anxiety Inventory
BDNF: Brain-derived neurotrophic factor
BMI: Body mass index
CRP: C-reactive protein
D-KEFS: The Delis-Kaplan Executive Function System
ECG: Electrocardiogram
EQ-5D: The EuroQoL-5 Dimension Questionnaire
GP: General practitioner
IGF-1: Insulin-like growth factor 1
M.I.N.I.: Mini International Neuropsychiatric Interview
MADRS: Montgomery Åsberg Depression Rating Scale
MTE: Metabolic equivalents
RCT: randomized controlled trial
RPE: Borg rated perceived exertion
VEGF: Vascular endothelial growth factor
VO2max: Maximal oxygen uptake capacity
WAIS-IV: The Wechsler Adult Intelligence Scale, 4th edition
