# Supplementary material for: Effects of exercise on symptoms of anxiety, cognitive ability and sick leave in patients with anxiety disorders in primary care: study protocol for PHYSBI, a randomized controlled trial
Source: BMC Psychiatry. 2019 Jun 10;19:172. doi: 10.1186/s12888-019-2169-5 (PMC6558952; doi:10.1186/s12888-019-2169-5)
Supplement: Supplementary file 1 — Questionnaire PHYSBI. (PDF 111 kb) [file 12888_2019_2169_MOESM1_ESM.pdf]

GÖTEBORGS UNIVERSITET

Questionnaire PHYSBI – a clinical interventional study of the importance of physical exercise on symptoms of anxiety, cognitive ability and work ability, in primary care.

1. **Name:**

2. **Personal identification number:**

3. **Date:**

4. **Marital state?**

   - ☐ Single
   - ☐ Married
   - ☐ Widowed
   - ☐ Divorced

5. **How old were you (in years) when you first had anxiety symptoms?**

6. **Do you have any other mental disorders? If yes, what diagnosis?**

7. **Do you have any other somatic disorders? If yes, what disorders?**

8. **Do you take any drugs/medicines at the moment? If yes, what are the names?**

9. **Do you take any drugs/medicines for mental illness? If yes:**
   What is the mane of the drug/medicine?
   What is the strength?
   How many pills per day?
   Have you taken this drug/medicine for longer than 4 weeks? (yes or No)
   What diagnosis do you take the drug/medicine for?

9. **Do you have ongoing psychotherapy? (Yes or No)**

10. **What is your highest education?**
   - ☐ Pre-high school education less than 9 years
   - ☐ Pre-high school education 9 years
   - ☐ High school education, maximum 2 years
   - ☐ High school education, 3 years
   - ☐ University education less than 3 years
   - ☐ University education 3 years or longer
   - ☐ Postgraduate education and research training

11. **What is your occupation?**

12. **What category describes your occupation:**
    - ☐ Higher official and senior position
    - ☐ Qualified official
    - ☐ Other official
    - ☐ Small entrepreneur excluding farmer
    - ☐ Farmer and similar
    - ☐ Forman and technician
    - ☐ Skilled worker within manufacturing, service and retail sectors
    - ☐ Skilled worker
    - ☐ Other worker
    - ☐ Not working

13. **How much do you work? How many percent of full-time (when/if you are not on sick leave)?**
    - ☐ 100%
    - ☐ ≥75%
    - ☐ ≥50%
    - ☐ ≥25%
    - ☐ I am unemployed
    - ☐ I am on maternal leave
    - ☐ I study

14. **Are you on sick leave? If yes, how much?**
    - ☐ 100%
    - ☐ ≥75%
    - ☐ ≥50%
    - ☐ ≥25%

15. **Do you currently do yoga?**

16. **Do you take any food supplements?**

17. **Do you smoke? If yes, how many cigarettes per day?**

18. **Level of physical activity before (1-2 years ago):** How many occasions and total minutes per week were you physically active so you got out of breath and/or started to sweat?
    Occasions per week:
    Total minutes per week:

19. **Current level of physical activity (last 2 months):** How many occasions and total minutes per week are you physically active so you get out of breath and/or start to sweat?
    Occasions per week:
    Total minutes per week:

20. Questing regarding diet

What type of diet do you usually eat? Mark one alternative, the most common!

☐ Mixed diet, eat most foods

☐ Only lactovegetarian, do not eat meat, fish or egg

☐ Mostly lactovegetarian, but sometimes eat fish and egg

☐ Vegan diet, do not eat meat, fish, egg, milk or dairy products

☐ Gluten free diet

☐ Other diet, describe;

How often do you eat the foods below? Mark average consumption the last year. Mark only one alternative for each category:

a) Fruit, berries and vegetables<br>☐ Never<br>☐ Once a month<br>☐ 1-3 times per month<br>☐ Once a week<br>☐ 2-3 times per week<br>☐ 4-6 times per week<br>☐ Once a day<br>☐ 2-3 times per day<br>☐ 4 times per day	b) Lean fish (cod, saithe, flatfish)<br>☐ Never<br>☐ Once a year<br>☐ 1-3 times per month<br>☐ Once a week<br>☐ 2-3 times per week<br>☐ 4-6 times per week<br>☐ Once a day<br>☐ 2-3 times per day<br>☐ 4 times per day	c) Fat fish (herring, salmon, mackerel)<br>☐ Never<br>☐ Once a year<br>☐ 1-3 times per month<br>☐ Once a week<br>☐ 2-3 times per week<br>☐ 4-6 times per week<br>☐ Once a day<br>☐ 2-3 times per day<br>☐ 4 times per day
d) Seafood (shrimps, crayfish, scollops)<br>☐ Never<br>☐ Once a year<br>☐ 1-3 times per month<br>☐ Once a week<br>☐ 2-3 times per week<br>☐ 4-6 times per week<br>☐ Once a day<br>☐ 2-3 times per day<br>☐ 4 times per day	e) Sweets including chocolate, candy, cookies, pastries)<br>☐ Never<br>☐ Once a year<br>☐ 1-3 times per month<br>☐ Once a week<br>☐ 2-3 times per week<br>☐ 4-6 times per week<br>☐ Once a day<br>☐ 2-3 times per day<br>☐ 4 times per day	f) Crisps, popcorn, salty nuts etc<br>☐ Never<br>☐ Once a year<br>☐ 1-3 times per month<br>☐ Once a week<br>☐ 2-3 times per week<br>☐ 4-6 times per week<br>☐ Once a day<br>☐ 2-3 times per day<br>☐ 4 times per day

g) French fries and/or pizza<br>☐ Never<br>☐ Once a year<br>☐ 1-3 times per month<br>☐ Once a week<br>☐ 2-3 times per week<br>☐ 4-6 times per week<br>☐ Once a day<br>☐ 2-3 times per day<br>☐ 4 times per day	h) Soft drinks, juice, lemonade, and/or fruit drinks<br>☐ Never<br>☐ Once a year<br>☐ 1-3 times per month<br>☐ Once a week<br>☐ 2-3 times per week<br>☐ 4-6 times per week<br>☐ Once a day<br>☐ 2-3 times per day<br>☐ 4 times per day	i) Coffee<br>☐ Never<br>☐ Once a year<br>☐ 1-3 times per month<br>☐ Once a week<br>☐ 2-3 times per week<br>☐ 4-6 times per week<br>☐ Once a day<br>☐ 2-3 times per day<br>☐ 4 times per day
j) Tea<br>☐ Never<br>☐ Once a year<br>☐ 1-3 times per month<br>☐ Once a week<br>☐ 2-3 times per week<br>☐ 4-6 times per week<br>☐ Once a day<br>☐ 2-3 times per day<br>☐ 4 times per day	k) What do you use on your sandwich?<br>☐ Bregott butter<br>☐ Butter<br>☐ Light maragrine<br>☐ Margarine	
