# Supplementary material for: Effects of exercise on symptoms of anxiety, cognitive ability and sick leave in patients with anxiety disorders in primary care: study protocol for PHYSBI, a randomized controlled trial
Source: BMC Psychiatry. 2019 Jun 10;19:172. doi: 10.1186/s12888-019-2169-5 (PMC6558952; doi:10.1186/s12888-019-2169-5)
Supplement: Supplementary file 2 — Work ability PHYSBI. (PDF 81 kb) [file 12888_2019_2169_MOESM2_ESM.pdf]

GÖTEBORGS UNIVERSITET

Work ability PHYSBI – a clinical interventional study of the importance of physical exercise on symptoms of anxiety, cognitive ability and work ability, in primary care.

1. **Assume that your workability at its best correspond to 10 point. What point would you give your current work ability?** Mark suitable number (0 means that you cannot work at all and 10 that your current work ability is at its best)

   *1 _ 2_ 3 _ 4 _ 5 _ 6 _ 7 _ 8 _ 9 _ 10_*

   Cannot work *My work ability is at its best*

2. **Have you during the last 12 months gone to work even though you, according to your health condition, should have taken sick leave?**
   Answer according to this scale:

   No, never
   Yes, once
   Yes, 2-5 times
   Yes, more than 5 times
